# Retinopathy of prematurity remains the leading cause of childhood blindness in children attending schools for the blind in Guadalajara: a fifteen-year comparison

**DOI:** 10.3389/fped.2026.1892954

**Published:** 2026-08-18

**Authors:** Luz Consuelo Zepeda Romero, Juan Carlos Barrera de Leon, ⁠José Alfonso Gutiérrez Padilla, Sofía Jacqueline Baeza Magaña, Ivana Jimenez Lopez, Sara Carolina Boyzo Arcadia, Mariana Padilla Escobar, Diana Estefanía Gutiérrez Gómez, Manuel Alejandro Del Callejo Bernal, Daniel Pérez Rulfo Ibarra, Guillermo Yanowsky Reyes

**Affiliations:** 1 Pediatric surgery – Hospital Civil de Guadalajara Unidad Hospitalaria Fray Antonio Alcalde, Guadalajara, Mexico; 2 Health Division – Universidad de Guadalajara – Centro Universitario de los Valles, Ameca, Mexico; 3 Mexican Federation of Neonatology A.C., Mexico; 4 Universidad de Guadalajara Centro Universitario de Ciencias de la Salud, Guadalajara, Mexico

**Keywords:** blindnes, children, screening criteria, neonatal care, prematurity, retinopathy of prematurity

## Abstract

**Objective:**

To identify the main causes of blindness in children attending at 3 schools for blind in the metropolitan area of Guadalajara (MAG), Jalisco, Mexico and to highlight the importance of preventing childhood visual disability.

**Methods:**

An observational, cross-sectional, and descriptive study was conducted in students enrolled in three schools for children with visual disabilities during the period September–December 2025 in Guadalajara, Jalisco, Mexico. Information was obtained from the review of clinical and school records, interviews with parents, and ophthalmologic evaluation, with prior institutional approval from the participating schools. A standardized World Health Organization (WHO) form was used to record sociodemographic variables, perinatal history, ophthalmologic diagnosis, etiology, treatments, and comorbidities. A descriptive analysis with an epidemiological approach was carried out using frequencies and measures of central tendency. Finally, the information was anonymized and handled confidentially, in accordance with ethical research principles.

**Results:**

This school-based, observational, descriptive cross-sectional study included a total of 106 children, 55 were female (51%) and 51 were male (49%). Retinal disorders constituted the main cause of visual loss with 64 cases (60%), predominantly sequelae of retinopathy of prematurity (ROP) in 50 children (47%). The second cause identified was optic nerve abnormalities with 19 cases (18%). 57 cases (54%) were associated with factors from the neonatal period. 50 children (47%) with diagnosis of ROP, had a mean gestational age of 30.6 weeks and a mean birth weight of 1443 g; 8 children (16%) were outside Mexican screening criteria and 28 (56%) outside United Kingdom criteria. 44 children (88%) with ROP presented blindness and 34 (68%) did not receive treatment and neurodevelopmental comorbidities were identified in 7 children (14%).

**Conclusions:**

Childhood blindness in the studied population was predominantly associated with potentially preventable causes, especially sequelae of ROP, positioning itself as the leading cause of childhood blindness in a school-based sample in Guadalajara. It is necessary to strengthen the prevention of retinopathy of prematurity through neonatal care, ensuring timely detection and treatment, as well as adapting neonatal screening programs and criteria to the regional context to reduce the burden of childhood visual impairment. These findings highlight the persistence of preventable ROP-related blindness among children attending specialized schools.

## Introduction

Vision allows human beings to interact with the environment and develop satisfactorily; therefore, its absence or deficiency generates consequences in different areas throughout life, having a special impact on children with delays in motor, linguistic, emotional, and social development, as well as academic performance (1).

According to the most current WHO estimates, approximately 19 million children under 15 years of age have some degree of visual disability and 1.4 million have irreversible blindness, of which 50% are due to preventable causes. The prevalence of blindness in children under 15 years of age in low and middle-income countries ranges from 0.2 to 7.8/10,000 people, and in developed countries the annual incidence is 6/10,000 (2). Current data on visual disability and childhood blindness available both worldwide and nationally are limited; the exact prevalence is unknown, making it difficult to understand the magnitude of the problem.

Epidemiological data regarding the causes of blindness in children vary considerably according to the region, conditioned by factors that affect healthcare quality, prevention, and screening in each population. Congenital cataracts are one of the main causes of visual disability in low-income countries; in middle-income countries such as Mexico, the main cause is Retinopathy of Prematurity (ROP), whereas cortical blindness is the principal cause in industrialized countries (1).

Epidemiological studies in schools for children with visual disabilities are a window of opportunity to learn more about the causes of blindness, providing an idea of deficiencies in visual healthcare within our context.

Guadalajara, located in western Mexico, is the second largest metropolitan area in the country, with an estimated population exceeding 5.3 million inhabitants. Mexico has a population of approximately 131 million people (3) and is classified by the United Nations Development Programme (UNDP) as a country with high human development (4). Life expectancy at birth is approximately 75 years, and primary school enrollment remains above 97%. In recent years, Mexico has reported nearly 2 million live births annually, with infant and under-5 mortality rates of approximately 12 and 14 deaths per 1,000 live births, respectively (5). These indicators suggest that the prevalence of childhood blindness is likely to be relatively low; however, retinopathy of prematurity (ROP) continues to represent an important and potentially preventable cause of childhood visual disability.

In 2010, a study was conducted in the city of Guadalajara, Jalisco, Mexico, in which the main causes of childhood blindness in schools for blind children were studied. A total of 144 children ≤15 years old with severe visual disability or blindness were included, ranging in age from 4 months to 15 years. In this population, ROP was identified as the most frequent cause of visual disability, responsible for 34.7% of cases (6).

Because more than a decade has passed since these observations, it is relevant to reevaluate the current causes of visual disability and childhood blindness together with the perinatal characteristics of children affected by ROP, in order to determine whether this condition continues to be the leading cause of childhood blindness in this school-based sample. Therefore, the objective of the present study is to identify the main causes of childhood blindness in 3 schools for children with visual disabilities in the Guadalajara Metropolitan Area, Jalisco, Mexico, and to describe the clinical and perinatal characteristics of cases associated with ROP.

## Methods

This is observational, descriptive, cross-sectional, school-based study conducted in 3 educational institutions for children with visual disabilities located in the Guadalajara Metropolitan Area, Jalisco, Mexico, during the period between August and December 2025. All children aged ≤15 attending the three centers who had severe visual impairment or blindness—defined as visual acuity of less than 6/60 in the better eye with the best possible correction (7) —were included.

Data collection was performed through interviews with parents, review of school and clinical records and ophthalmologic examination carried out by ophthalmologists measuring visual acuity with the “E” test. For this purpose, a standardized data extraction form was designed and used to systematically record the variables of interest. The collected information included sociodemographic data, perinatal history, ophthalmologic diagnosis, etiology of visual disability, treatments received, and associated comorbidities.

The WHO system (6, 8) was used to classify the causes of blindness and low vision in children. This system uses two methods for reporting causes of blindness and low vision in children; the first is a classification according to the affected anatomical site: whole globe, cornea, lens, uvea, retina, optic nerve, glaucoma, and conditions in which the eye appears normal. The second classifies according to the underlying etiology: hereditary, intrauterine, perinatal, childhood, and unknown.

The obtained data were organized in a database for statistical analysis. A descriptive analysis was carried out through calculation of absolute and relative frequencies, as well as measures of central tendency and dispersion for continuous variables. Results were presented in tables and figures to facilitate interpretation, with an epidemiological approach aimed at describing the distribution of causes of visual disability and their main characteristics in the studied population.

The information was handled confidentially and anonymized, without including data that would allow participant identification.

## Results

The study population consisted of 110 children with visual disabilities. A total of 106 schoolchildren ≤15 years old from three institutions (51, 45, and 10 students, respectively) were included; four were excluded for exceeding the age limit. In one of the participating institutions (10 children), information was provided through an institutional list that included the names and ophthalmologic diagnoses of the students and parents telephone interviews; however, examination of those children was restricted. Of the total, 51% were female and 49% were male, of whom 73% presented blindness. A total of 75% came from the Guadalajara Metropolitan Area, the remaining 25% originated from municipalities outside the Guadalajara Metropolitan Area, including rural areas of Jalisco and neighboring states.

The anatomical distribution of the identified causes of visual loss, stratified by age group and compared with the study conducted in 2010 by Zepeda (6) and collaborators, is shown in Table 1.

**Table 1:** Anatomical site and etiological classification of visual loss by age group.

Anatomical site	0−4 years *N* (%)	5−9 years *N* (%)	10−15 years *N* (%)	Total 2025 *N* (%)	Total 2010 *N* (%)
Retina				**64** **(60)**	69 (48)
Retinopathy of prematurity^a^	10 (20)	17 (34)	23 (46)	50 (47)	50 (35)
Retinal dystrophy	1 (14)	4 (57)	2 (29)	7 (6)	8 (6)
Retinoblastoma	0 (0.00)	1 (25)	3 (75)	4 (4)	3 (2)
Retinal detachment	0 (0.00)	2 (75)	1 (25)	3 (3)	3 (2)
Coloboma	0 (0.00	0 (0.00)	0 (0.00)	0 (0.00)	5 (4)
Optic nerve	3 (16)	6 (32)	10 (53)	**19** (**18)**	25 (17)
Glaucoma	0 (0.00	1 (25)	3 (75)	**4** (**4)**	21 (15)
Whole globe				**8** (**7)**	13 (9)
Microphthalmos	1 (17)	3 (50)	2 (33)	6 (6)	8 (6)
Anophthalmos	1 (50)	1 (50)	0 (0.00)	2 (1)	5 (4)
Normal ocular globe^b^	1 (17)	4 (67)	1 (17)	**6** (**5)**	9 (6)
Cornea	0 (0.00)	0 (0.00)	0 (0.00)	**0** (**0.00)**	3 (2)
Lens	0 (0.00)	1 (50)	1 (50)	**2** (**2)**	2 (1)
Other	0 (0.00)	2 (75)	1 (25)	**3** (**3)**	2 (1)
				106 (100)	144 (100)

Bold values highlights the overall values for main anatomical sites and overall total population for the 2025 cohort, subcategories represent specific etiologies within each site.

a Includes stage 5 retinopathy of prematurity and cicatricial changes.

b Includes refractive error, amblyopia and cortical visual impairment.

Retinal disorders were the most frequent in 64 children (60%), specifically sequelae of ROP being the main cause in 50 children (47%). In second place were optic nerve lesions in 19 children (18%), followed by disorders involving the whole globe in 8 children (7%) and congenital glaucoma in 4 children (4%). By age group, retinopathy of prematurity accounted for visual disability in 20 children younger than 5 years (20%), 17 children aged 5–9 years (34%), and 23 children aged 10 years or older (46%) (Table 1).

Ethologically, most cases were associated with neonatal factors, accounting for 57 children (54%). Conditions of uncertain onset were identified in 24 children (22%), hereditary causes in 19 children (18%), intrauterine causes in 4 children (4%), and causes acquired in childhood in 2 children (2%) (Table 2).

**Table 2:** Etiological factors.

Factors	*N* (%)
Hereditary	19 (18)
Intrauterine	4 (4)
Neonatal	57 (54)
Childhood	2 (2)
Unknown	24 (22)

**Table 3:** Details of children with visual loss from retinopathy of prematurity in schools for the blind in Guadalajara city.

Characteristic	Total 2010	Total 2025
Gestational age (weeks), mean (range)	28 (25−34)	30.6 (24.5−35)
Birth weight (g), mean (range)	1,200 (700−1,980)	1,443.74 (800−2,295)
GA and BW outside UK criteria	5 (10%)	28 (56%)
GA and BW outside Mexican criteria	0 (0%)	8 (16%)
Sex		
Male	20 (40%)	26 (52%)
Female	30 (60%)	24 (48%)
History of AAG, laser or VR surgery	23 (46%)	16 (32%)
Degree of visual loss		
Severe visual impairment	10 (20%)	6 (12%)
Blindness	40 (80%)	44 (88%)
Other impairment		
Psychomotor delay	39 (78%)	5 (10%)
Physical impairment	8 (16%)	1 (2%)
Hearing loss	1 (2%)	0 (0%)
Learning difficulties	1 (2%)	4 (8%)
Other		7 (14%)
None		32 (64%)
Total	50 (100%)	50 (100%)

Values are *N* (%) unless otherwise stated.

**Figure 1:**
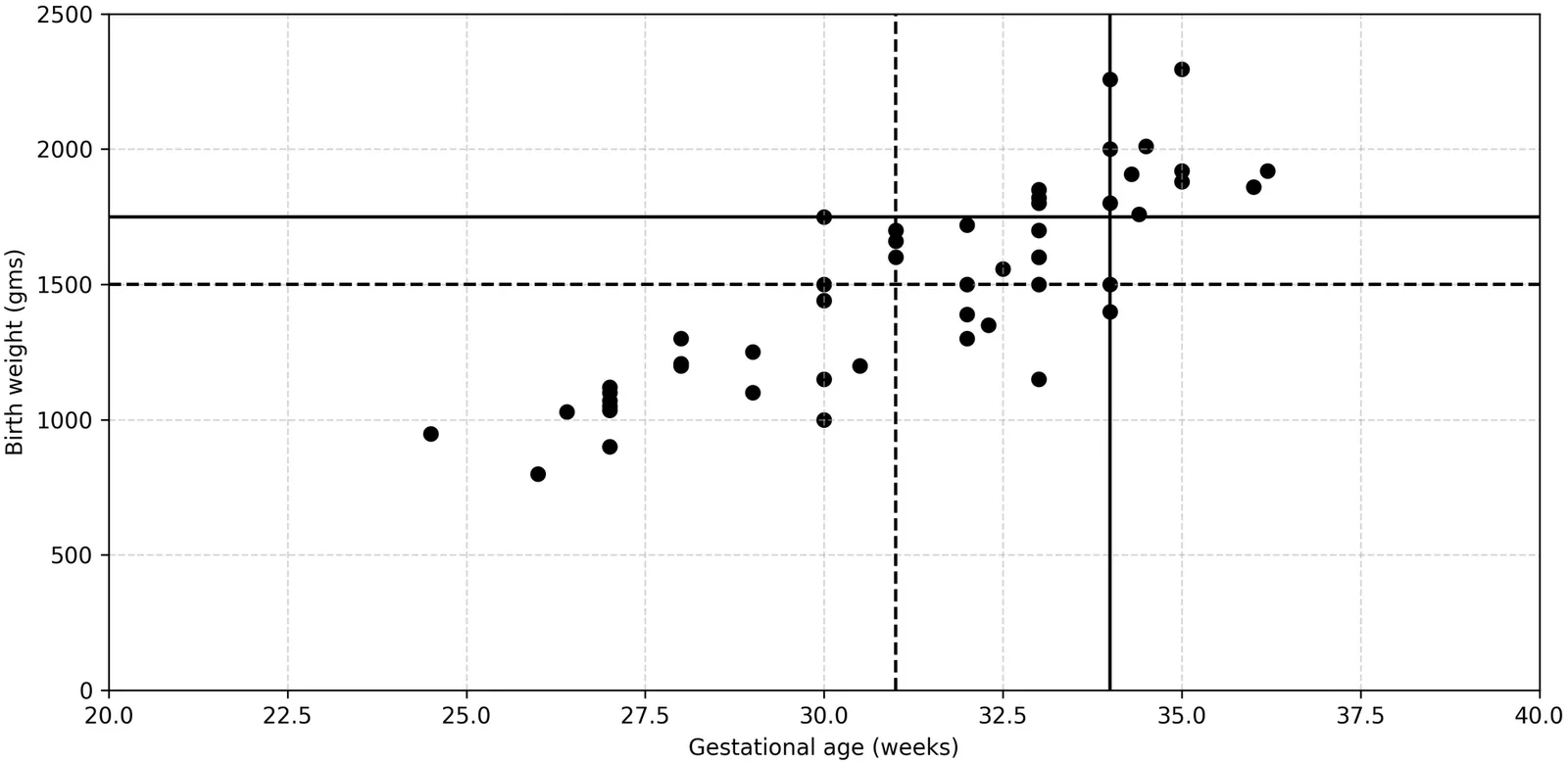
Birth weight and gestational age of children with visual loss due to retinopathy of prematurity (ROP). The continuous vertical line at ≤34 gestational age and horizontal line at ≤1750g indicate the criteria used in the Mexican national guidelines for ROP programs, while the dashed vertical line at ≤31 gestational age and the horizontal line at ≤1500 g indicate the UK national ROP screening criteria.

The mean gestational age of children with ROP was 30.6 weeks (range: 24.5–35) and the mean birth weight was 1443.74 g (range: 800–2295 g). A total of 56% presented combinations of birth weight and gestational age outside United Kingdom screening criteria, while under the Mexican criteria only 16% fall outside the established screening range (Figure 1).

Regarding treatment, only 16 children (32%) had a history of laser photocoagulation, antiangiogenic therapy, or vitreoretinal surgery. Blindness occurred in 44 children (88%), The remaining 6 children (12%) have severe visual disability. Among associated comorbidities, psychomotor delay was identified in 5 children (10%), learning difficulties in 4 children (8%), physical disability in 1 child (2%), and others in 7 children (14%). In 32 children (64)%, no other alterations were recorded (Table 3).

## Discussion

The results show predominance of retinal diseases as the main cause of childhood blindness among children included in this school-based sample, particularly sequelae of retinopathy of prematurity (ROP). This finding is consistent with reports from contexts where neonatal survival has increased, neonatal intensive care services access services (NICUs) have expanded without homogeneous implementation of adequate oxygen administration, screening, and timely treatment programs for ROP (16), which may contribute to the persistence of this pathology as the principal preventable cause of visual disability in contries like our. Suboptimal neonatal care predisposes larger and more mature infants to develop severe and aggressive posterior ROP, a phenomenon described as the third ROP epidemic (9).

When comparing the results of the present study with those reported in 2010 (6), differences in the distribution of causes of childhood visual disability were observed. The retina disorder was the most frequently affected anatomical site in both studies, however, its proportion increased from 60% of cases in the present study compared with 47.9% in 2010. ROP accounted for a higher proportion, from 34.7% in 2010 to 47% in 2025, being the most frequent individual etiology of visual disability in both school-based samples. In contrast, other causes such as glaucoma represented a lower proportion of cases in the present study than in 2010, 4% vs. 14.6% respectively. Although hereditary and postnatal causes were less frequent, they remain important from an epidemiological perspective.

Analysis of the subgroup of children with ROP, a higher proportion of children presented characteristics outside the screening criteria established in the United Kingdom, which include newborns <31 weeks gestational age or with birth weight <1501 g (10), these findings suggest that their use does not fit the local epidemiological profile of this school-based samples. This has important implications, since incorrect application of international criteria could result in exclusion of patients at risk of developing ROP and favor delayed diagnosis.

In Mexico, since 2012, screening has been regulated by Article 61 of the General Health Law and the Clinical Practice Guideline, which indicate that screening be performed starting at the fourth week of life in neonates ≤34 weeks gestational age and/or birth weight ≤1750g, as well as in larger or older neonates with associated risk factors, according to the treating physician’s judgment (11, 12). Although Mexican criteria include a broader at-risk population, 16% of children with ROP in the present sample were outside these criteria, mainly due to their birth weight, highlighting the importance of applying gestational age as an independent screening criterion, regardless of birth weight.

In 84% of cases that did meet the Mexican screening criteria that were not detected and treated in time, resulting in some degree of visual impairment or blindness, could be related for the lack of ophthalmologists and inadequate healthcare infrastructure, this leads to uneven coverage of screening and treatment, thereby constituting a cause of advanced-stage in middle-income settings such as the one in this school-based sample (13).

In terms of treatment, the 2010 study reported that 54% of children with ROP received treatment; among the population evaluated in 2025, treatment was reported in a lower proportion of cases (32%). Despite technological advances and greater knowledge about the disease, barriers to referral mechanisms and access to specialized ophthalmological care may persist.

Concerning systemic sequelae, in 2010 a higher proportion presented psychomotor developmental delay (78%), whereas a lower proportion was observed in the 2025 study (10%).

With respect to the origin of the children included in the present study, 25% came from outside the Guadalajara Metropolitan Area, including rural areas of Jalisco and neighboring states, in contrast to the 2010 study, in which 16% belonged to this group.

Nevertheless, the results of this study should be interpreted considering its limitations. Being a cross-sectional design, it prevents establishment of causal relationships between perinatal factors and the appearance of blindness. The study population was drawn exclusively from schools for children with visual disabilities, which limits the generalization of the findings to all children with visual impairments in the general population, particularly those who do not have access to the formal educational system and may have different clinical characteristics. It was not possible for direct ophthalmologic examination in one of the participating institutions, and information for these children was obtained from institutional records and parental interviews, which may have resulted in some degree of misclassification. Finally, perinatal history and treatment information were partially obtained from parental recall, which may be subject to recall bias. Despite these limitations, the inclusion of the three main institutions for children with visual disabilities in the Guadalajara Metropolitan Area provides valuable insight into the current causes of childhood blindness among children included in this school-based sample.

## Conclusion

Taken together, these findings indicate that ROP was the most frequently identified cause among children enrolled in schools for the blind in Guadalajara, Jalisco, Mexico, highlighting the need to strengthen prevention, screening, follow-up, and timely treatment strategies during the neonatal period. NICUs should be accompanied by appropriate ROP screening programs and access to specialized ophthalmologic care for at-risk infants.

The findings supportS the need to adapt ROP screening programs to the regional context, guarantee timely ophthalmologic evaluation of at-risk newborns, and ensure follow-up until complete retinal vascularization (15). Likewise, the identification of cases outside international criteria highlights the importance of adhering to national guidelines and carefully applying gestational age when determining eligibility for screening.

These strategies, together with continuous improvement of prenatal and neonatal care, healthcare personnel training, and timely access to treatments, are fundamental to reduce the burden of preventable childhood visual disability associated with ROP.

## Data Availability

The de-identified data supporting the findings of this study are available from the corresponding author upon reasonable request, subject to applicable ethical and institutional requirements.
